# Possible Involvement of Differential Ubiquitination as a Molecular Basis of Phenotypic Heterogeneity in Neurodevelopmental Disorders

**DOI:** 10.3390/genes17050553

**Published:** 2026-05-05

**Authors:** Tadashi Nakagawa, Makiko Nakagawa

**Affiliations:** 1 Department of Clinical Pharmacology, Faculty of Pharmaceutical Sciences, Sanyo-Onoda City University, Sanyo-Onoda 756-0084, Yamaguchi, Japan; 2 Institute of Gene Research, Yamaguchi University Science Research Center, Ube 755-8505, Yamaguchi, Japan; mnakagaw@yamaguchi-u.ac.jp; 3 Advanced Technology Institute, Life Science Division, Yamaguchi University, Yamaguchi 755-8611, Yamaguchi, Japan

**Keywords:** neurodevelopmental disorders, phenotypic heterogeneity, ubiquitination, UBE3A, CUL5-ARIH2, NEDD4L, histone H2A, β-catenin, MeCP2, SHANK3

## Abstract

Neurodevelopmental disorders (NDDs) are characterized by remarkable phenotypic heterogeneity, in which individuals harboring mutations in the same gene display divergent clinical manifestations, ranging from mild cognitive impairment to severe neurodevelopmental deficits. Advances in neurogenetics and neurogenomics have rapidly expanded the catalog of genes associated with NDDs and have provided unprecedented insight into the genetic architecture of these conditions. However, how identical or similar genetic variants give rise to such diverse phenotypic outcomes remains largely unknown. Ubiquitin-mediated protein regulation is a central mechanism controlling diverse processes essential for neural development, including chromatin regulation, transcriptional dynamics, protein turnover, and synaptic function. Importantly, ubiquitination is a multilayered regulatory process governed by multiple determinants, including the availability of ubiquitination sites on substrates, the activity of ubiquitin ligases, the opposing actions of deubiquitinases, and priming post-translational modifications such as phosphorylation or acetylation. These regulatory layers create a dynamic ubiquitination landscape that may vary across individuals, cell types, developmental stages, and environmental contexts. In this review, we discuss how insights from neurogenetics and neurogenomics can be integrated with knowledge of ubiquitin signaling to better understand the molecular basis of phenotypic heterogeneity in NDDs. We propose that differential ubiquitination represents an important mechanistic framework through which genetic variation is translated into diverse molecular and cellular outcomes. Understanding the interplay between neurogenetic variation and ubiquitin-dependent regulatory networks may provide new perspectives on disease mechanisms and inform future therapeutic strategies for neurodevelopmental disorders.

## 1. Introduction

Neurodevelopmental disorders (NDDs) encompass a diverse group of conditions characterized by impairments in cognitive, behavioral, and social functions that arise from disrupted brain development [1,2]. Advances in neurogenetics and neurogenomics have dramatically expanded our understanding of the genetic architecture underlying these disorders, such as the identification of numerous causative or risk-associated genes [3,4].

A striking feature of NDDs is the remarkable phenotypic heterogeneity observed among affected individuals [5,6,7]. Patients carrying identical or highly similar genetic variants frequently exhibit widely divergent clinical manifestations, ranging from mild cognitive impairment to severe intellectual disability or autism spectrum-related phenotypes. This variability poses a major challenge for both mechanistic understanding and clinical management, and the molecular principles that translate genetic variation into diverse phenotypic outcomes remain largely unresolved. One possible explanation for such phenotypic diversity lies in the multilayered regulatory networks that control protein function in developing neurons. Among these regulatory systems, ubiquitin-mediated protein modification has emerged as a central mechanism governing diverse aspects of cellular physiology, including protein stability, signal transduction, and transcriptional regulation [8,9,10,11].

**Figure 1:**
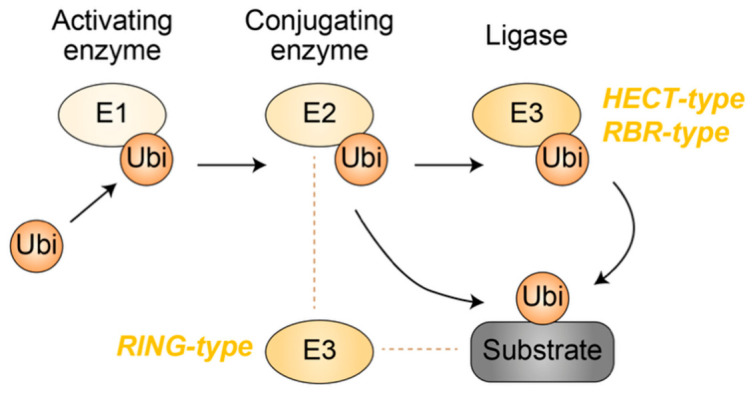
Protein ubiquitination mediated by a multistep enzymatic cascade. E1 ubiquitin-activating enzymes form a high-energy thioester bond with ubiquitin. E2 ubiquitin-conjugating enzymes subsequently accept ubiquitin from E1. HECT- and RBR-type E3 ubiquitin ligases receive ubiquitin from E2 and catalyze its transfer to substrate proteins. In contrast, RING-type E3 ubiquitin ligases function by bridging ubiquitin-charged E2 enzymes and substrate proteins, thereby facilitating direct ubiquitin transfer. Yellow denotes ubiquitin or enzymes involved in ubiquitination. Grey represents the substrate. Arrows indicate the directionality of ubiquitin transfer. Ubi, ubiquitin.

Protein ubiquitination is a multistep enzymatic cascade in which ubiquitin is covalently attached to substrate proteins through the sequential actions of E1 ubiquitin-activating enzymes, E2 ubiquitin-conjugating enzymes, and E3 ubiquitin ligases (Figure 1). In the first step, E1 enzymes initiate the ubiquitin conjugation cascade by forming a high-energy thioester bond between the active-site cysteine of the enzyme and the C-terminus of ubiquitin in an ATP-dependent manner [12]. Activated ubiquitin is subsequently transferred to the active-site cysteine of an E2 enzyme [13]. In the final step, E3 ubiquitin ligases mediate the transfer of ubiquitin from the E2 enzyme to substrate proteins, thereby conferring substrate specificity to the ubiquitination process [14,15].

E3 ubiquitin ligases are broadly classified into three major families based on their structural features and mechanisms of ubiquitin transfer: really interesting new gene (RING), homologous to E6AP C-terminus (HECT), and RING–IBR–RING (RBR) ligases [14,15] (Figure 1). RING-type ligases function primarily as scaffolds that juxtapose ubiquitin-charged E2 enzymes and substrate proteins, thereby facilitating the direct transfer of ubiquitin from E2 to the substrate. In contrast, HECT- and RBR-type ligases employ a catalytic cysteine residue to form an E3–ubiquitin thioester intermediate prior to ubiquitin transfer. In RBR ligases, the RING1 domain recruits the ubiquitin-loaded E2 enzyme, whereas the RING2 domain contains the catalytic cysteine responsible for ubiquitin transfer to the substrate. Recent comprehensive annotations estimate that the human genome encodes 302–420 RING-type, 28 HECT-type, and 14 RBR-type E3 ubiquitin ligases [16,17].

A substantial proportion of E3 ubiquitin ligases function as multi-subunit complexes composed of scaffold proteins, adaptor molecules, and interchangeable substrate receptors, often accompanied by additional regulatory subunits. Prototypical examples include Cullin-RING ubiquitin ligases (CRLs), which are organized around a cullin scaffold (e.g., CUL1, CUL2, CUL3, CUL4A/B, and CUL5), a RING protein (RBX1 or RBX2), adaptor proteins (such as SKP1 for CRL1, Elongin B/C for CRL2 and CRL5, and DDB1 for CRL4 complexes), and diverse substrate receptors (including F-box proteins for CRL1, SOCS-box proteins for CRL2 and CRL5, BTB proteins for CRL3, and DCAF family members for CRL4) [18,19]. Another representative multi-subunit E3 complex is the anaphase-promoting complex/cyclosome (APC/C), a large ubiquitin ligase comprising approximately 20 subunits, including a cullin-like subunit (APC2), a RING subunit (APC11), and co-activators such as Cdc20 and Cdh1 that mediate substrate recognition [20,21]. Recent comprehensive annotations estimate that the human genome encodes approximately 73, 32, 97, 49, and 53 substrate receptors for CRL1, CRL2, CRL3, CRL4, and CRL5, respectively [17].

The activity of CRLs is further regulated by conjugation of the ubiquitin-like protein NEDD8, a process termed neddylation [18,19]. Similar to ubiquitination, neddylation proceeds through an enzymatic cascade that culminates in the covalent attachment of the C-terminal glycine of NEDD8 to a conserved lysine residue on cullin proteins. Neddylation induces conformational rearrangements within the CRL complex, promoting an open and dynamic architecture that enhances the proximity between ubiquitin-loaded E2 enzymes and substrate proteins, thereby stimulating ubiquitin transfer. Conversely, the removal of NEDD8 by the COP9 signalosome (CSN) facilitates the binding of CAND1, which promotes the exchange of substrate receptors and remodeling of CRL complexes [22]. Thus, the dynamic cycles of neddylation and deneddylation are essential for regulating CRL activity and enabling the efficient targeting of a broad spectrum of substrates.

Protein ubiquitination changes a protein’s stability, localization, interactions, and activity, depending on how many ubiquitins are attached and which linkages are used. The canonical function of ubiquitination is to induce substrate degradation. In this case, K48-linked polyubiquitin chains, formed through successive ubiquitination of the ε-amino group of lysine 48 on ubiquitin, serve as a degradation signal recognized by the 26S proteasome, which subsequently unfolds and translocates substrates into its proteolytic core [23,24]. In contrast, monoubiquitination on membrane proteins promotes endocytosis and sorting into multivesicular bodies, leading to the downregulation of receptors and channels at the plasma membrane [25,26]. Another well-studied function is mediated by K63-linked ubiquitin chains that create docking platforms for ubiquitin-binding domains, assembling or disassembling signaling complexes in pathways such as NF-κB, DNA damage response, and innate immunity [27].

**Figure 2:**
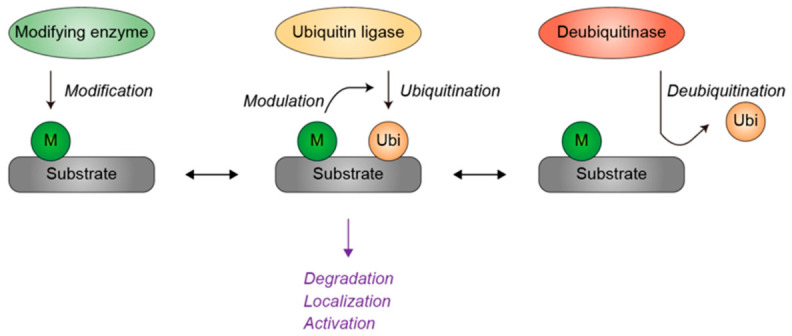
Regulation of ubiquitination by substrate modification, ubiquitin ligases, and deubiquitinases. Protein-modifying enzymes, such as kinases and acetyltransferases, modify substrate proteins, thereby modulating their susceptibility to subsequent ubiquitination. Deubiquitinases remove ubiquitin moieties from substrates, thereby counteracting the activity of ubiquitin ligases. Ubiquitination can result in proteasomal degradation, altered subcellular localization, or the modulation of protein activity. Green indicates substrate modifications or modifying enzymes. Yellow denotes ubiquitin or ubiquitin ligases. Red represents deubiquitinases. Grey denotes the substrate. Single-headed arrows indicate the directionality of the modification, whereas double-headed arrows represent equilibrium. M, modification. Ubi, ubiquitin.

Importantly, ubiquitination is also not a simple on-off modification but rather a highly dynamic process regulated at multiple levels. The extent of substrate ubiquitination is determined by several factors, including the availability of lysine residues on substrate proteins, the activity and specificity of ubiquitin ligases, the opposing actions of deubiquitinases (DUBs), and priming post-translational modifications such as phosphorylation or acetylation that influence ubiquitin conjugation (Figure 2) [28,29,30,31]. These regulatory layers generate a flexible ubiquitination landscape that may vary among cell types, developmental stages, and environmental contexts. Consequently, even subtle differences in these regulatory mechanisms may alter the ubiquitination status of key substrates, leading to divergent molecular and cellular consequences.

In the nervous system, ubiquitin signaling has been shown to play essential roles in neural progenitor proliferation, neuronal differentiation, and synapse formation [32,33,34,35]. Consistent with these functions, mutations in components of the ubiquitin system have been increasingly linked to NDDs through neurogenetic studies. In this context, combined with the above-described multilayered regulation of ubiquitination, we propose that differential ubiquitination of critical neuronal substrates may represent an important molecular mechanism contributing to phenotypic heterogeneity in NDDs. Rather than focusing broadly on the ubiquitin system, this review highlights representative examples in which multiple regulatory layers converge on specific substrates relevant to NDDs. We discuss UBE3A, a representative ubiquitin ligase strongly associated with neurodevelopmental disorders, histone H2A ubiquitination, one of the most abundant modifications in the nervous system, β-catenin, a central signaling molecule in Wnt pathways, methyl-CpG binding protein 2 (MeCP2), a transcriptional regulator implicated in Rett syndrome, and SH3 and multiple ankyrin repeat domains 3 (SHANK3), a synaptic scaffold protein strongly associated with autism spectrum disorders. In addition, we discuss CUL5-ARIH2, SENP8, and NEDD4L as representatives that are the recently identified gene products associated with ubiquitination defects and NDDs. Through these examples, we illustrate how the convergence of ubiquitin ligases, DUBs, and priming post-translational modifications at the level of individual substrates may generate differential ubiquitination states, potentially contributing to the phenotypic diversity observed in patients with NDDs.

## 2. UBE3A as a Paradigm of Differential Ubiquitin Ligase Activity in Neurodevelopmental Phenotypes

No gene more clearly exemplifies database-informed genotype–phenotype stratification than UBE3A (OMIM #105830 for Angelman syndrome; OMIM #608636 for 15q11–q13 duplication syndrome), the sole gene within the 15q11–q13 locus that is consistently expressed exclusively from the maternal allele in mature neurons [36,37,38]. The striking clinical divergence between Angelman syndrome (AS), characterized by hypersociability, ataxic gait, and microcephaly, and 15q11–q13 duplication syndrome (Dup15q), marked by autistic behaviors and hypotonia without microcephaly, is interpreted as a direct consequence of opposing alterations in UBE3A ubiquitin ligase activity [39].

**Figure 3:**
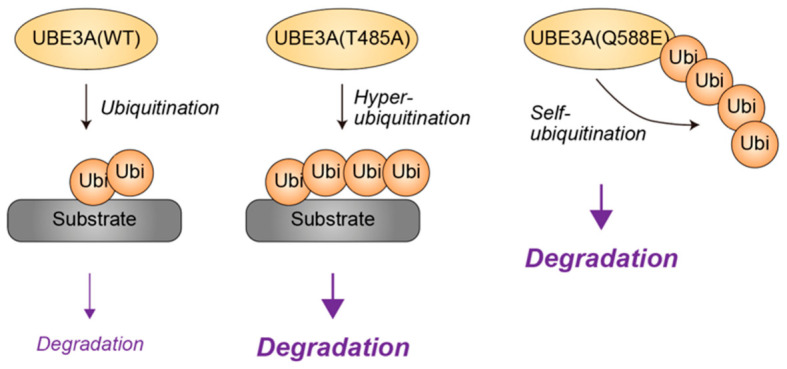
Differential ubiquitin ligase activity of UBE3A driven by NDD-associated missense mutations. UBE3A harboring the T485A mutation [UBE3A(T485A)] exhibits approximately 1.8-fold higher ubiquitin ligase activity than wild-type UBE3A [UBE3A(WT)], resulting in hyper-ubiquitination of substrate proteins and enhanced substrate degradation. In contrast, the Q588E variant [UBE3A(Q588E)] displays an approximately fivefold increase in ubiquitin ligase activity, promoting pronounced autoubiquitination and consequent proteasomal degradation of UBE3A itself. Yellow denotes ubiquitin or ubiquitin ligases. Grey represents the substrate. Black arrows indicate the directionality of ubiquitin transfer, while purple arrows denote the functional consequences of ubiquitination, with arrow thickness reflecting the magnitude of the effect. Ubi, ubiquitin.

Notably, ClinVar catalogs hundreds of UBE3A missense variants, approximately two-thirds of which are classified as variants of uncertain significance (VUS) [40]. A comprehensive functional interrogation of 152 non-truncating UBE3A variants, predominantly annotated as VUS, revealed that approximately 12% represent hyperactivating gain-of-function (GOF) alleles rather than loss-of-function mutations [40]. Seventeen individuals carrying such hyperactivating variants exhibited neurodevelopmental phenotypes, including intellectual disability, autism spectrum features, and seizures, which overlap with but remain clinically distinguishable from AS. Consistent with these observations, murine models harboring the UBE3A GOF variant T503A (corresponding to human T485A), which enhances enzymatic activity by approximately 75% relative to wild-type levels, display reduced steady-state UBE3A protein levels due to enhanced auto-ubiquitination, concomitant depletion of substrate proteins, and behavioral phenotypes distinct from AS models [41]. In contrast, mice expressing the more potent GOF variant Q606E (human Q588E), which increases enzymatic activity by approximately 388%, exhibit phenotypes closely resembling AS, accompanied by the accumulation of UBE3A substrates, likely reflecting excessive auto-ubiquitination and near-complete depletion of UBE3A protein [42] (Figure 3). Furthermore, a mouse model carrying additional copies of murine Ube3a, resulting in a threefold increase in UBE3A protein abundance and reduced substrate levels, recapitulates autistic-like phenotypes analogous to those observed in Dup15q syndrome [43].

Collectively, these findings underscore that differential ubiquitination dynamics including auto-ubiquitination of the ligase itself drive neurodevelopmental phenotypic heterogeneity through nuanced modulation of ubiquitin ligase abundance and catalytic activity. Importantly, these insights have immediate implications for ClinVar interpretation: hyperactivating UBE3A variants have historically been classified as VUS due to a pathogenicity framework predicated primarily on loss-of-function (LOF) mechanisms. Integrating high-resolution functional data with curated genetic databases is therefore essential to accurately resolve phenotypic heterogeneity arising from gain-of-function ubiquitination.

## 3. CUL5-ARIH2 Ubiquitin Ligase as an Allosteric E3-E3 Hub for Differential Ubiquitination

**Figure 4:**
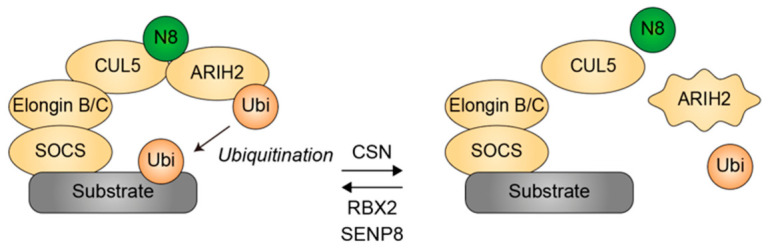
Regulation of the E3-E3 ubiquitin ligase CUL5–ARIH2 by neddylation. RBX2 conjugates NEDD8 to CUL5, generating neddylated CUL5. Neddylated CUL5 engages ARIH2, which provides catalytic activity for the ubiquitination of substrate proteins recruited to CUL5 via the Elongin B/C adaptor complex and one of 53 SOCS box-containing substrate receptors. Removal of NEDD8 from CUL5 by the COP9 signalosome (CSN) dissociates the substrate adaptor–receptor–substrate assembly as well as ARIH2, resulting in catalytic inactivation of the CUL5-based ubiquitin ligase. This process also promotes an autoinhibited conformation of ARIH2, as indicated by distortion of the outer frame. SENP8 provides free NEDD8 for attachment to CUL5. Yellow denotes ubiquitin or ubiquitin ligases. Green indicates NEDD8. Grey represents the substrate. Arrows in the left panel indicate the directionality of ubiquitin transfer, whereas arrows between panels represent equilibrium, with the corresponding enzymes involved depicted above or below each arrow. N8, NEDD8. Ubi, ubiquitin.

CUL5-ARIH2 represents a paradigmatic E3-E3 ubiquitin ligase module in which allosteric, NEDD8-dependent activation and substrate routing provide a mechanistic framework for differential ubiquitination and its contribution to NDD phenotypic heterogeneity. Within this system, CUL5 functions as a scaffold of CUL5 Cullin-RING ligases (CRL5s), recruiting substrates via SOCS adaptor proteins [34], whereas ARIH2, an RBR-type E3 ligase, remains autoinhibited until activated by association with RBX2-catalyzed neddylated CUL5 [44]. Upon activation, ARIH2 provides catalytic capacity for ubiquitin chain initiation and elongation, thereby shaping ubiquitin architecture on specific lysine residues of substrate proteins [45,46] (Figure 4). Since ARIH2 is capable of catalyzing both K48- and K63-linked polyubiquitin chains, including mixed K48/K63 chains [47], this E3-E3 configuration might allow the same CRL5-substrate complex to generate distinct ubiquitin chain types and topologies depending on ARIH2 engagement, thereby exemplifying the principle of differential ubiquitination.

In this context, SENP8 emerges as a critical higher-order regulator of the CUL5–ARIH2–NEDD8 axis. SENP8 is a NEDD8-specific protease that processes NEDD8 precursors and removes NEDD8 from aberrantly modified components of the neddylation machinery, thereby maintaining pathway fidelity [48]. Although SENP8 does not seem to efficiently deneddylate CUL5 directly [48], it exerts indirect control over CUL5 activity by preserving the free NEDD8 pool for CUL5 neddylation. Consistently, loss of SENP8 results in the accumulation of aberrantly neddylated intermediates, the reduced availability of functional E2 enzymes such as UBE2F, and consequently impaired CUL5 neddylation and CRL5 activity [48].

Importantly, SENP8 expression in neurons is developmentally regulated, peaking around the first postnatal week and declining in the mature brain, coinciding with periods of robust neurite outgrowth and synapse formation [49]. Perturbation of SENP8 alters actin dynamics, Wnt/β-catenin signaling, autophagy, and excitatory synapse maturation, supporting a broad role for this deneddylase in modulating neuronal development via neddylation-related mechanisms [49]. Given that CRLs represent the principal downstream effectors of NEDD8, these observations strongly implicate SENP8 as a rheostat controlling CRL5–ARIH2 activity in the developing nervous system.

Integration of SENP8 into the differential ubiquitination framework reveals multiple mechanistic layers. First, SENP8 governs the “activation window” of the CUL5–ARIH2 complex by modulating the proportion of productively neddylated CUL5 assemblies capable of engaging ARIH2. Subtle variations in SENP8 expression or activity can therefore shift the equilibrium between RING-only ubiquitination and full E3-E3 cooperative activity, thereby altering ubiquitin chain length and topology on shared substrates. Second, SENP8 interfaces with pathways such as Wnt/β-catenin signaling and cytoskeletal regulation, which overlap with CRL-dependent ubiquitination networks, thereby indirectly influencing substrate selection and modification patterns. Third, the temporal dynamics of SENP8 expression introduce a developmental dimension whereby modest perturbations during critical windows may elicit disproportionately large effects on neural circuit formation.

Although, to the best of our knowledge, mutations in the CUL5 or RBX2 genes have not been reported in patients with NDDs, LOF variants in a substrate receptor, WSB2, have been identified [50], and a probable LOF mutation in the ARIH2 gene has also recently been described [51]. Given the limited number of affected individuals, it remains unclear whether these mutations contribute to phenotypic heterogeneity in NDDs through differential ubiquitination of substrate proteins. Furthermore, NEDD8 substrates beyond Cullin family proteins are increasingly recognized as playing critical roles in neurodevelopment and neurological disease [52], raising the possibility that differential neddylation also contributes to the phenotypic heterogeneity of NDDs.

## 4. NEDD4L as a HECT-Type Ubiquitin Ligase Driving Differential Ubiquitination

NEDD4L (also known as NEDD4-2) is emerging as a paradigmatic example of how differential ubiquitination mediated by a single E3 ligase can generate pronounced phenotypic heterogeneity in neurodevelopmental disorders. NEDD4L is a HECT-type ubiquitin ligase characterized by an N-terminal C2 domain, multiple WW domains that mediate substrate recognition, and a C-terminal catalytic HECT domain that directly transfers ubiquitin to substrates [53,54]. It is broadly expressed, including in cortical progenitors, and plays pivotal roles in neuronal migration, cortical patterning, and the regulation of cellular excitability [54,55,56].

**Figure 5:**
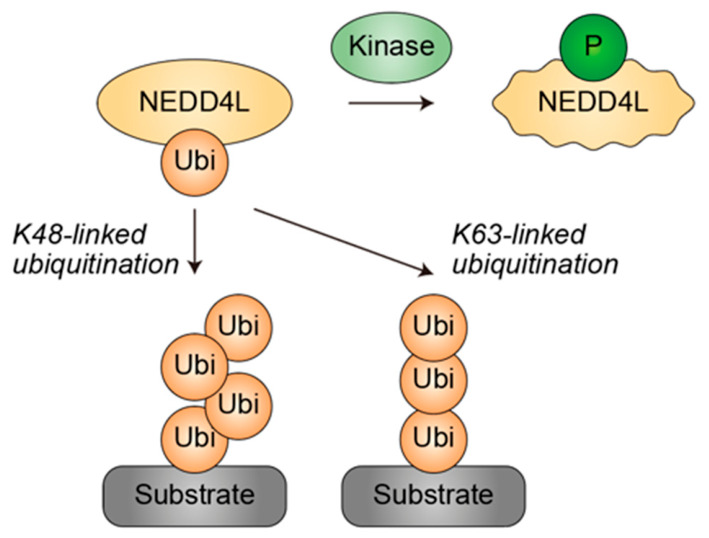
Phosphorylation-dependent regulation of the HECT-type ubiquitin ligase NEDD4L and its capacity to generate diverse ubiquitin chain topologies. Phosphorylation promotes the inactivation of NEDD4L, as illustrated by distortion of the outer frame. NEDD4L catalyzes multiple ubiquitin linkage types, including K48- and K63-linked chains. Yellow denotes ubiquitin or ubiquitin ligases. Green indicates kinases and phosphorylation. Grey represents the substrate. Arrows indicate the directionality of modifications. P, phosphate group. Ubi, ubiquitin.

A defining feature of NEDD4L biology is its capacity to assemble diverse ubiquitin chain topologies. Rather than being restricted to canonical K48- and K63-linked chains, NEDD4L can catalyze multiple linkage types, including K6-, K11-, and K33-linked chains [57]. These distinct ubiquitin codes encode diverse functional outcomes, ranging from proteasomal degradation to the modulation of signaling pathways, subcellular localization, and protein–protein interactions [58,59]. Accordingly, NEDD4L exemplifies the principle of differential ubiquitination, whereby a single ligase can direct multiple, context-dependent fates for its substrates. This functional versatility is further refined by intramolecular autoinhibition: interactions among the C2, WW, and HECT domains [57,60,61], together with phosphorylation-dependent binding of 14-3-3 proteins [62,63], modulate the conformation of NEDD4L, toggling it between closed, autoinhibited and open, catalytically active states (Figure 5).

In the developing brain, NEDD4L intersects with multiple signaling pathways critical for neurodevelopment. These include Wnt signaling via ubiquitination of Dishevelled2 (Dvl2) [64], AKT–mTOR signaling [55], and inflammatory signaling through MEKK2 [65]. Each of these pathways is highly dosage-sensitive, and even modest perturbations in ubiquitination can profoundly influence progenitor proliferation, neuronal migration, and synaptic maturation. Consequently, alterations in NEDD4L activity can propagate through multiple regulatory networks, amplifying their impact on cortical development.

Genetically, pathogenic variants in NEDD4L are associated with periventricular nodular heterotopia type 7 (PVNH7) (OMIM #617201), a malformation of cortical development characterized by aberrant neuronal migration and structural cortical abnormalities [66]. Notably, disease-associated missense variants predominantly cluster within the HECT domain, suggesting that altered catalytic activity, rather than defective substrate recognition, is a principal driver of pathogenesis [55,67,68,69]. Despite this shared molecular basis, reported PVNH7 cases exhibit pronounced intra- and inter-familial phenotypic variability, ranging from isolated bilateral periventricular nodular heterotopia with mild developmental delay to severe neurodevelopmental impairment accompanied by refractory epilepsy and complex cortical malformations [55,66,67,68,69].

This phenotypic heterogeneity may be mechanistically attributable to differential ubiquitination. First, HECT-domain mutations may differentially perturb ubiquitin chain initiation, elongation, and linkage specificity, resulting in substrate-specific alterations in degradation or signaling, as reported for other HECT-type ubiquitin ligases [70,71,72,73]. Second, competition among substrates may bias ubiquitination toward specific pathways depending on cellular context, developmental stage, or modifier factors. Third, interactions with DUBs and priming post-translational modifications further modulate substrate ubiquitination, introducing additional layers of regulatory complexity. Finally, tissue- and stage-specific expression patterns ensure that identical molecular perturbations can give rise to distinct phenotypic outcomes across developmental contexts and organ systems.

## 5. Ubiquitination of H2A

Histone H2A monoubiquitination at lysine 119 (H2AK119ub1) is a fundamental chromatin modification catalyzed by Polycomb Repressive Complex 1 (PRC1) [74]. PRC1 comprises a structurally heterogeneous family of multisubunit E3 ubiquitin ligase complexes, whose catalytic core consists of a RING1A/RING1B heterodimer associated with the one of six PCGF (PCGF1-6) scaffold and several other accessory proteins. Canonical PRC1 (cPRC1; PCGF2/4-CBX-PHC) is recruited to loci marked by H3K27me3 deposited by Polycomb Repressive Complex 2 (PRC2), whereas non-canonical PRC1 (ncPRC1; PCGF1/3/5/6-RYBP/YAF2) preferentially targets CpG islands independently of PRC2 [75]. H2AK119ub1 is removed by several DUBs, most notably the Polycomb Repressive DUB (PR-DUB) complex composed by BAP1 (the catalytic DUB subunit) and one of three ASXL (ASXL1-3) [74,76].

During neural development, H2AK119ub1 establishes repressive chromatin at developmental regulators, orchestrating the proliferation and differentiation of neural stem/progenitor cells [77,78]. Consistently, germline mutations in PRC1 writer components (RING1A, RING1B, PCGF2, PHC1, BCORL1) and PR-DUB erasers (BAP1 and ASXL1-3) give rise to a growing family of syndromic NDDs, all converging on dysregulation of H2AK119ub1 homeostasis [79,80,81,82].

**Figure 6:**
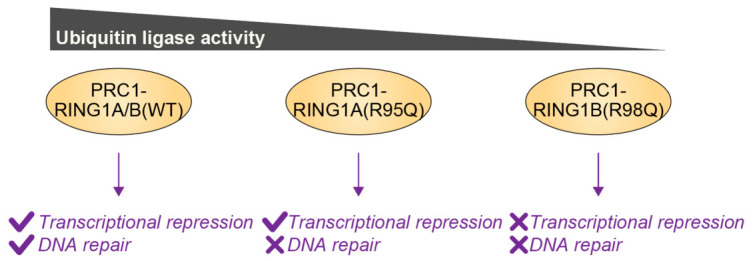
Differential effects of PRC1 ubiquitin ligase activity on transcriptional repression and DNA repair. PRC1 complexes containing wild-type (WT) RING1A or RING1B mediate both transcriptional repression and DNA repair. In contrast, PRC1 harboring the NDD-associated RING1A R95Q variant [RING1A(R95Q)] exhibits an approximately 36% reduction in ubiquitin ligase activity relative to WT PRC1 and displays impaired DNA repair while largely preserving transcriptional repression. A more pronounced defect is observed in PRC1 containing the RING1B R98Q variant [RING1B(R98Q)], which retains approximately half of the WT activity and exhibits deficiencies in both transcriptional repression and DNA repair. Gray triangle indicates relative ubiquitin ligase activity. Yellow denotes ubiquitin ligases. Purple denotes the functional consequences associated with each PRC1 complex, with check marks indicating functional activity and cross marks denoting impairment.

Recent studies employing human embryonic stem cells engineered to harbor patient-derived dominant negative variants in RING1A (R95Q) [83] and corresponding RING1B (R98Q) have revealed graded reductions in H2AK119ub1 levels: RING1A^R95Q/+^ decreases H2AK119ub1 by 33%, RING1A^R95Q/R95Q^ by 36%, and RING1B^R98Q/R98Q^ by 48% [84]. Notably, the more pronounced depletion observed in RING1B^R98Q/R98Q^ cells induces extensive transcriptional dysregulation and delayed DNA repair, whereas the more modest reduction in RING1A^R95Q/R95Q^ neural progenitor cells impairs DNA repair without eliciting widespread transcriptional derepression or differentiation defects (Figure 6). In both contexts, defective DNA repair leads to S-phase stalling and perturbed cell cycle dynamics, thereby linking PRC1 dysfunction to neurodevelopmental phenotypes such as microcephaly [84]. These findings suggest that DNA repair processes exhibit greater sensitivity to reduction in H2AK119ub1 than transcriptional repression, providing the example that differential ubiquitination impacts distinct cellular functions.

Given that components of the Polycomb Repressive DUB complex, namely BAP1 and ASXL1–3, are frequently mutated in patients with neurodevelopmental disorders (NDDs), and that cells derived from individuals harboring ASXL3 mutations exhibit elevated H2AK119ub1 levels [85], it is plausible that excessive accumulation of H2AK119ub1 contributes to disease pathogenesis. Intriguingly, contrary to the conventional paradigm in which increased H2AK119ub1 enforces transcriptional repression of Polycomb Repressive Complex 1 target genes, loss of BAP1 leads to aberrant intergenic spreading of H2AK119ub1, resulting in inappropriate activation of these targets [86,87]. Moreover, BAP1 is recruited to sites of DNA damage, and its localization inversely correlates with H2AK119ub1 levels; depletion of BAP1 leads to excessive accumulation of H2AK119ub1 at DNA lesions [88]. Dynamic cycles of ubiquitination and deubiquitination of H2AK119ub1 at damage sites are proposed to finely regulate chromatin accessibility: H2AK119ub1 facilitates the initiation of the DNA damage response and transcriptional silencing, whereas BAP1-mediated removal locally relaxes chromatin to promote DNA end resection and the recruitment of DNA repair factors [89,90]. Elucidating how variations in DNA repair efficiency and transcriptional repression intersect with NDD pathogenesis represents a critical avenue for understanding the molecular basis of disease heterogeneity.

## 6. Ubiquitination of β-Catenin

β-Catenin is a central component of the Wnt signaling pathway, which plays essential roles in neural development, including the regulation of neural progenitor proliferation, neuronal differentiation, and synapse formation [91,92,93]. Consistent with these functions, dysregulation of β-catenin signaling has been implicated in NDDs, and mutations in the gene encoding β-catenin, *CTNNB1*, have been identified in patients with intellectual disability, autism spectrum disorder, and other developmental abnormalities [94,95]. A notable feature of *CTNNB1*-associated disorders is the considerable variability in clinical manifestations among affected individuals [96,97], suggesting that regulatory mechanisms controlling β-catenin activity may modulate disease phenotypes.

The abundance and activity of β-catenin are tightly regulated by ubiquitin-mediated proteolysis [98]. In the absence of Wnt signaling, β-catenin is incorporated into a multiprotein destruction complex composed of Axin, adenomatous polyposis coli (APC), glycogen synthase kinase 3 (GSK3), and casein kinase 1 (CK1). Within this complex, β-catenin undergoes sequential phosphorylation by CK1 and GSK3 at specific N-terminal residues. These phosphorylation events generate a phosphodegron that is recognized by the SCF^β-TrCP^ ubiquitin ligase complex, which catalyzes the polyubiquitination of β-catenin and targets it for degradation by the 26S proteasome. Through this mechanism, phosphorylation acts as a priming modification that enables ubiquitin-mediated turnover of β-catenin. Binding of Wnt ligands to their receptors leads to inhibition of the destruction complex, resulting in the stabilization and accumulation of β-catenin. Stabilized β-catenin interacts with TCF/LEF transcription factors to activate gene expression programs that control cell proliferation and differentiation. The ubiquitination status of β-catenin therefore acts as a molecular switch that determines the activity of Wnt signaling.

In addition to ubiquitin ligases, DUBs contribute to the dynamic regulation of β-catenin stability. Four DUBs (i.e., USP2a, USP4, USP6NL, and USP47) have been reported to remove ubiquitin chains from β-catenin, thereby promoting its stabilization and enhancing Wnt signaling activity [99,100,101,102]. Although pathogenic mutations in the genes encoding these DUBs have not yet been identified, all four DUBs are expressed in the brain [103,104,105,106]. Notably, *Usp2* gene knockout mice exhibit an anxiety-like behavior [105], a phenotype frequently observed in patients with NDDs. Combined with the report demonstrating that excessive expression of β-catenin in the brain induces autism-like behaviors in mice [107], these observations suggest that the activity or abundance of these DUBs may influence NDD pathogenesis through β-catenin regulation.

**Figure 7:**
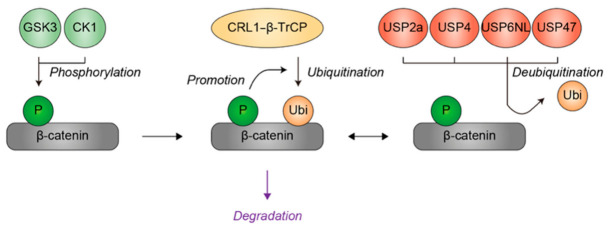
Regulation of β-catenin ubiquitination by substrate phosphorylation, ubiquitin ligase, and deubiquitinases. GSK3 and CK1 phosphorylate β-catenin, thereby promoting its susceptibility to subsequent ubiquitination catalyzed by the CRL1 ubiquitin ligase complex containing β-TrCP as the substrate receptor (CRL1–β-TrCP). USP2a, USP4, USP6NL, and USP47 remove ubiquitin from β-catenin, thereby antagonizing ubiquitin-mediated degradation. Green indicates phosphate group or kinases. Yellow denotes ubiquitin or the CRL1–β-TrCP ubiquitin ligase complex. Red represents deubiquitinases. Grey represents the β-catenin. Black arrows indicate the directionality of the modifications, while purple denotes the functional consequence of ubiquitination. CRL1, Cullin-RING ubiquitin ligase 1. P, phosphate group. Ubi, ubiquitin.

Taken together, β-catenin provides a well-characterized example in which multi-layered regulation of substrate ubiquitination determines signaling output (Figure 7). Variations in kinase activity, expression levels of ubiquitin ligases or DUBs, or cellular signaling contexts may shift the threshold at which β-catenin is degraded or stabilized, and such differences could lead to divergent transcriptional responses during neurodevelopment, even in individuals carrying similar genetic variants affecting β-catenin signaling. Given the central role of Wnt signaling in brain development, differential ubiquitination of β-catenin may represent a plausible mechanism contributing to the phenotypic heterogeneity observed in NDDs.

## 7. Ubiquitination of MeCP2

MeCP2 is a transcriptional regulator that plays a crucial role in neuronal maturation [108]. It binds methylated CpG dinucleotides in DNA and modulates gene expression through interactions with chromatin remodeling complexes and other transcriptional regulators [109,110]. The importance of MeCP2 in neurodevelopment is underscored by the fact that mutations in the *MeCP2* gene cause Rett syndrome, a severe NDD characterized by intellectual disability, motor impairment, and autistic features [111,112]. Notably, both loss-of-function and increased dosage of MeCP2 can result in neurological abnormalities, highlighting the requirement for the precise regulation of MeCP2 abundance and activity [113,114].

Consistently, emerging evidence indicates that MeCP2 is subject to multiple post-translational modifications that may regulate its stability [115]. Phosphorylation at S92 by HIPK2 and S413 by an unknown kinase is positioned within PEST degrons and has therefore been postulated to promote MeCP2 degradation [115,116], although this mechanism has not yet been experimentally validated. Several ubiquitin ligases (i.e., RNF4, NEDD4, HERC2, and CRL4–DCAF13) ubiquitinate MeCP2 and promote its proteasomal degradation, thereby contributing to the turnover of this transcriptional regulator [117,118,119,120]. In contrast, the deubiquitinase USP15 counteracts this ubiquitin-mediated degradation by removing ubiquitin moieties from MeCP2 [121]. Interestingly, mutations in genes encoding CUL4B, a core scaffold component of the CRL4 complex, and USP15 have been identified in patients with NDDs. Determining whether these patients exhibit altered MeCP2 abundance will be of important in future studies. If confirmed, these findings would suggest that differential ubiquitination of MeCP2 contributes to NDD pathogenesis in these individuals.

**Figure 8:**
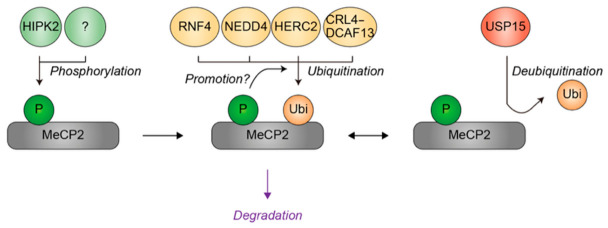
Regulation of MeCP2 ubiquitination by substrate phosphorylation, ubiquitin ligases, and a deubiquitinase. HIPK2 and an unidentified kinase phosphorylate MeCP2 within PEST degrons, likely enhancing its susceptibility to subsequent ubiquitination catalyzed by RNF4, NEDD4, HERC2, and the CRL4 ubiquitin ligase complex containing DCAF13 as the substrate receptor (CRL4–DCAF13). USP15 removes ubiquitin from MeCP2, thereby antagonizing ubiquitin-mediated degradation. Green indicates phosphorylation events or kinases. Yellow denotes ubiquitin or the ubiquitin ligases. Red represents a deubiquitinase USP15. Grey represents the MeCP2. Black arrows indicate the directionality of the modifications, while purple denotes the functional consequence of ubiquitination. A question mark denotes a kinase that remains to be identified. CRL4, Cullin-RING ubiquitin ligase 4. P, phosphate group; Ubi, ubiquitin.

Given that both excessive and reduced levels of MeCP2 can lead to NDDs, variability in the regulatory mechanisms controlling MeCP2 ubiquitination may contribute to the broad phenotypic spectrum observed in disorders associated with MeCP2 dysfunction (Figure 8). In this regard, extracellular cues such as synaptic activity and neuromodulatory signals have been shown to converge on nuclear MeCP2 through Ca^2+^-dependent and cAMP/PKA-dependent kinase pathways, inducing site-specific phosphorylation that modulates MeCP2 chromatin binding and its interactions with transcriptional cofactors [122,123]. In parallel, MeCP2 ubiquitination in neurons is also likely governed by intracellular signaling cascades downstream of these extracellular stimuli. However, key mechanistic aspects, including the specific E3 ubiquitin ligases involved, the lysine residues targeted for ubiquitination, and the stimulus–response kinetics, remain poorly defined in the neuronal literature. Taken together, differential ubiquitination of MeCP2 provides an illustrative example of how multilayered regulation of transcriptional regulators may contribute to phenotypic heterogeneity in NDDs.

## 8. Ubiquitination of SHANK3

Ubiquitination of synaptic proteins is widely recognized as a dynamic regulatory mechanism that shapes synapse formation, stability, and multiple forms of synaptic plasticity [124,125]. Among these substrates, SHANK3 is a prominent core component of the postsynaptic density (PSD), where it orchestrates the assembly of multiprotein complexes linking neurotransmitter receptors, signaling molecules, and cytoskeletal elements [126,127]. Genetic studies have established that mutations or deletions of the SHANK3 gene are strongly associated with autism spectrum disorders and Phelan–McDermid syndrome, underscoring the importance of SHANK3 in normal brain development and synaptic function [128,129]. Notably, individuals carrying similar SHANK3 variants often display considerable variability in clinical severity [130,131], suggesting that regulatory mechanisms affecting SHANK3 stability may influence phenotypic outcomes.

The abundance of SHANK3 at synapses is tightly controlled by protein turnover mechanisms. ERK2 phosphorylates SHANK3 at three serine residues (S1134, S1163, and S1253), resulting in enhanced ubiquitination and subsequent degradation of SHANK3 [132]. Although the ubiquitin ligases responsible for SHANK3 ubiquitination remain to be characterized, USP8 has been identified as a deubiquitinase that removes ubiquitin chains from SHANK3, thereby promoting its stabilization [133].

**Figure 9:**
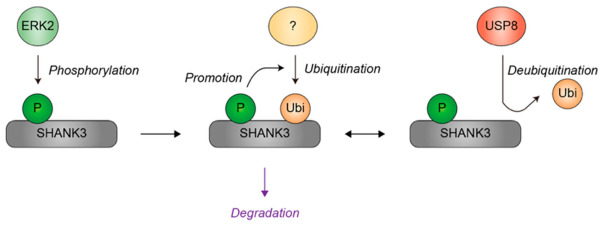
Regulation of SHANK3 ubiquitination by substrate phosphorylation, ubiquitin ligase(s), and a deubiquitinase. ERK2 phosphorylates SHANK3, thereby enhancing its susceptibility to subsequent ubiquitination catalyzed by currently unidentified ubiquitin ligase(s). USP8 removes ubiquitin from SHANK3, thereby antagonizing ubiquitin-mediated degradation. Green indicates phosphorylation events or kinases. Yellow denotes ubiquitin or ubiquitin ligases. Red represents the deubiquitinase USP8. Grey represents the SHANK3. Black arrows indicate the directionality of the modifications, while purple denotes the functional consequence of ubiquitination. A question mark denotes a ubiquitin ligase that remains to be identified. P, phosphate group; Ubi, ubiquitin.

Given the central role of SHANK3 in organizing synaptic architecture, alterations in its stability or turnover may exert profound effects on synaptic development and plasticity. Variability in the regulatory pathways controlling SHANK3 ubiquitination could therefore influence synaptic protein homeostasis, potentially contributing to the diverse phenotypic manifestations observed among individuals carrying SHANK3 mutations (Figure 9). As with MeCP2, extracellular events such as synaptic activity and ischemic stress have been shown to regulate SHANK3 protein levels through ubiquitin-dependent proteasomal degradation in neurons [133,134], indicating that environmental conditions can modulate the ubiquitination and degradation of SHANK3. In this manner, SHANK3 exemplifies how differential ubiquitination of synaptic proteins may constitute a molecular mechanism underlying phenotypic heterogeneity in NDDs.

## 9. Conclusions

Advances in neurogenetics and neurogenomics have markedly expanded the repertoire of genes associated with NDDs. Nevertheless, a central challenge that remains unresolved is the striking phenotypic heterogeneity observed among patients carrying identical or closely related genetic variants. The molecular mechanisms that translate genetic variation into diverse cellular and clinical outcomes are therefore an important subject of investigation.

In this review, we have highlighted key ubiquitin ligases and neuronal substrates as a potential mechanism contributing to such phenotypic variability. Ubiquitination is regulated through multiple interconnected layers, including substrate accessibility, ubiquitin ligases, DUBs, and priming post-translational modifications. Through the integration of these regulatory processes, the ubiquitination status of a given substrate can vary substantially depending on cellular context. As illustrated by the examples of β-catenin, MeCP2, and SHANK3, such multilayered regulation converges at the level of individual proteins that control signaling pathways, transcriptional programs, and synaptic organization, respectively. Variability in these regulatory mechanisms may therefore influence the stability or activity of these substrates and ultimately shape neurodevelopmental outcomes.

Importantly, recent neurogenetic and neurogenomic studies increasingly reveal that many NDD-associated genes encode proteins involved in ubiquitin signaling or its regulatory networks. Integrating these genetic insights with molecular studies of ubiquitination dynamics may provide a valuable framework for understanding how genetic variants are translated into heterogeneous phenotypes. In particular, multi-omics approaches that combine genomic, transcriptomic, and proteomic analyses at multiple time points in different cell types may help uncover context-dependent ubiquitination states of disease-relevant substrates across different neuronal cell types and developmental stages.

Current evidence strongly suggests, yet falls short of definitively establishing, a causal relationship between differential ubiquitination of specific substrates and the nature or severity of NDD phenotypes. It is well-recognized that NDD-associated phenotypes exhibit substantial variability even among model organisms harboring identical genetic modifications and maintained under uniform environmental conditions. This variability may, at least in part, be attributable to the differential ubiquitination of substrate proteins implicated in NDD pathogenesis. To rigorously establish causality, it will be essential to elucidate the spatiotemporal regulation of ubiquitination dynamics, including the finely tuned activity of ubiquitin ligases, DUBs, and other post-translational modifiers that collectively determine the ubiquitination levels. As highlighted throughout this review, such nuanced regulatory mechanisms are likely to play a critical role in shaping phenotypic heterogeneity and must be systematically investigated to delineate their contribution to NDD pathophysiology.

Another important topic to be investigated is the crosstalk between ubiquitination and epigenetic mechanisms, particularly histone methylation. There are at least three mechanistic layers of crosstalk: (1) ubiquitin on histones directly modulates H3 methylation [135,136], (2) ubiquitination controls the stability and localization of histone methyl writers/erasers [137], and (3) methyl marks can in turn regulate ubiquitin ligases and DUBs to reinforce or switch epigenetic states [138]. In essence, ubiquitination does not just coexist with histone methylation, but it actively configures the methylation landscape, creating recruitment platforms for chromatin complexes, which can be the causes and consequences of differential ubiquitination.

Although many questions remain regarding the precise mechanisms by which ubiquitin signaling contributes to NDD pathogenesis, a ubiquitin ligase- and substrate-centered perspective may provide a useful conceptual framework. By focusing on how multiple regulatory layers converge on key neuronal proteins, it becomes possible to consider how subtle variations in ubiquitination dynamics might amplify or buffer the effects of genetic mutations. Further investigation into these regulatory networks may therefore not only improve our understanding of phenotypic heterogeneity in NDDs, but also provide new opportunities for therapeutic strategies aimed at modulating ubiquitin-dependent pathways in an individual patient-specific manner.

## Data Availability

No new data were created or analyzed in this study. Data sharing is not applicable to this article.
